# Risk and protective factors related to changes in mental health among adolescents since COVID-19 in Hong Kong: a cross-sectional study

**DOI:** 10.1186/s13034-023-00622-x

**Published:** 2023-06-12

**Authors:** Cheuk Yui Yeung, Vera Yu Men, Wendy W. Y. So, Daniel Yee Tak Fong, Mona Wai Cheung Lam, Derek Yee Tak Cheung, Paul Siu Fai Yip

**Affiliations:** 1 Department of Social Work and Social Administration, The University of Hong Kong, Pokfulam, Hong Kong SAR China; 2 Hong Kong Jockey Club Centre for Suicide Research and Prevention, The University of Hong Kong, Hong Kong SAR, China; 3 School of Nursing, Li Ka Shing Faculty of Medicine, The University of Hong Kong, Hong Kong SAR, China; 4 The Family Planning Association of Hong Kong, Hong Kong SAR, China

**Keywords:** Mental health, COVID-19, Psychiatric epidemiology, Public health, Adolescents

## Abstract

**Background:**

Most research has suggested that children and adolescents had poorer mental health than pre-COVID-19 pandemic status. There have been few investigations into factors associated with pre-peri pandemic differences in young people’s mental health status. Our study aimed to investigate the association between sociodemographic factors, attitudes, and daily life experiences and these differences.

**Methods:**

We used self-reported cross-sectional data from the Youth Sexuality Survey (YSS) by the Family Planning Association of Hong Kong, collected from secondary school students aged 10–16 between the fourth and fifth waves of the pandemic. The study outcome was pre-peri pandemic differences in mental health (better, unchanged, or poorer). Associations between the study outcome with age, sex, satisfaction with academic performance, school life, relationship with classmates and family life, and average sleeping and exercising time in the past month, were assessed through multinomial logistic regression, controlling for depressive/anxiety symptoms and change in physical health status since the pandemic.

**Results:**

There were 6,665 respondents. Compared with pre-pandemic, approximately 30% reported poorer mental health, whilst 20% reported better mental health. Females (OR = 1.355, 95% CI = 1.159–1.585) and those dissatisfied with their academic performance (OR = 1.468, 95% CI = 1.233–1.748) were significantly more likely to report poorer mental health with reference to unchanged status, while those satisfied with family life had improved mental health with reference to unchanged (OR = 1.261, 95% CI = 1.006–1.579) and poorer status (OR = 1.369, 95% CI = 1.085–1.728).

**Conclusion:**

Policy and community strategies that promote good family relationships are thus essential for young people’s mental health during societal challenges such as the COVID-19 pandemic.

**Supplementary Information:**

The online version contains supplementary material available at 10.1186/s13034-023-00622-x.

## Background

The coronavirus disease (COVID-19) pandemic had severe physical, economic, social and emotional impacts on nearly every country in the world. While young people infected with COVID-19 were reported to suffer relatively mild physical symptoms with a good prognosis, there are greater concerns about the broader impact of the pandemic on their mental health regardless of their infection status [1]. Previous study suggested that large-scale lockdowns and social distancing restrictions may disrupt their normal routines and networks, through the closure of schools and other places of socialization such as restaurants, cinemas, and sports facilities, as well as stay-safe-at-home recommendations [2].

The COVID-19 pandemic first hit Hong Kong in January 2020. Until the end of 2022, Hong Kong experienced five waves of the pandemic. The daily number of confirmed cases of COVID-19 from the first confirmed case until the end of 2022 is shown in Appendix I [3]. The number of cases in the first two years of the pandemic remained at a relatively low number. The number increased drastically during February and March in 2022 as the Omicron variant presented in Hong Kong [4].

In response to Government-mandated isolation policies, schools in Hong Kong were closed for most of 2020, for the first few months in 2021, and again for a few months in 2022. During school closure, teaching activities were conducted online. Even after reopening of schools, different measures were implemented including but not limited to wearing face masks at all times, half-day school times to avoid having lunch at school, cancellation of extra-curricular and other populous school activities, etc. [5] School-aged children had limited social interactions and physical activities, which have been reported around the world as detrimental to their physical and mental well-being [6, 7]. However, it is possible that some young people may have benefited from COVID-19 pandemic related policies, as has been reported in a small amount of international literature, and there have been none in the Hong Kong context [8–10]. Previous studies in the United States (US) and Canada suggested that although as much as 70% of adolescents reported poorer mental health, a certain proportion of them reported improvement (19-31% in the Canadian study and 5.4% in the US) [8, 9]. What is more, research to date around the world has not examined whether factors that are known to be important to young people’s mental health like school experiences and daily life activities (e.g., exercise, sleeping, etc.) are related to changes in pre-peri pandemic mental health [2].

This acts as an exploratory study which aims to identify patterns of change in mental health status among young people in Hong Kong pre-peri COVID-19 pandemic, and to investigate the association between sociodemographic factors, attitudes and daily life experiences and the change in mental health status.

## Methods

### Data

The Family Planning Association of Hong Kong conducted the Youth Sexuality Survey (YSS) from May to July 2021 (between the fourth [November 2020 to April 2021] and fifth [January 2022 onwards] wave of the pandemic) among secondary school students in Hong Kong [4]. The daily number of confirmed cases in 2021 and the data collection period are depicted in Appendix II [3]. A list of all government-aided, caput or direct subsidized full-time day schools (N = 455) was obtained from the Education Bureau of the Hong Kong Special Administrative Region Government. Students from non-Chinese-speaking and evening schools were not involved. Stratified random sampling was conducted by four areas in Hong Kong (Hong Kong Island, Kowloon Peninsula, New Territories West, and New Territories East), and selected schools (N = 304) were invited to take part in the survey. The survey was conducted by a supervised self-administrated approach. An informed consent written in Chinese was provided to the parents introducing the purpose of the survey. It also stated that the participation in the survey was completely voluntary and anonymous. Twenty-five schools participated and the overall response rate was 78.1% with a total of 8,343 respondents.

This study utilized a subset of the respondents aged between 10 and 16 years old, since the definitions of a child and a young person in Hong Kong were “under the age of 14 years” and “14 years of age or upwards and under the age of 16 years” respectively [11]. Most of the respondents in the YSS were within 10–16 years (84.2%).

### Measures

The study outcome was a single survey item that asked respondents *“what changes in mental health have you encountered since COVID-19”* with five options (much poorer, poorer, unchanged, better and much better). For analysis purposes, those who responded much poorer or poorer, or better or much better were considered to have “poorer” or “better” mental health, respectively.

Apart from age and sex, other variables of interest included participants’ satisfaction with their academic performance, school life, relationship with classmates and family life. These variables were each measured as single items with five responses (very dissatisfied, dissatisfied, neutral, satisfied and very satisfied). Answers of very dissatisfied or dissatisfied, satisfied or very satisfied were collapsed into “dissatisfied” and “satisfied”, respectively.

Average sleeping time and exercise time in the past month over weekdays and weekends were also captured. For sleeping time, the responses categories were “less than 7 hours”; “7–9 hours”; or “more than 9 hours”, based on the definition of sleep deprivation for people younger than 18 [12]. Exercise time was categorized into “less than 30 minutes” and “more than or equal to 30 minutes” [13].

Four items related to depressive/anxiety symptoms in the past two weeks from PHQ-2 and GAD-2 were included as control variables [14, 15]. Respondents were asked about the frequency of having any of four symptoms: (i) feeling nervous, anxious or on edge; (ii) not being able to stop or control worrying; (iii) little interest or pleasure in doing things and (iv) feeling down, depressed or hopeless. Four options were provided, namely none, somedays, half of the days or nearly every day. Change in physical health status because of COVID-19 was also included as a control variable. It was measured similarly to the mental health status. Respondents were grouped into “poorer”, “unchanged” and “better”.

### Data analytic strategy

ANOVA and Chi-squared test were performed to compare continuous and categorical characteristics, respectively between the three categories of respondents: unchanged, poorer and better mental health. Differences in profiles of varying self-reported mental health status during COVID-19 were assessed by a multinomial logistic regression. The regression analyses included all the variables of interest. The percentage of missing values among these variables ranged from 0.0 to 6.9%. Multiple imputation was performed to account for missing values among the covariates. Adjusted odds ratios (aORs) with 95% confidence intervals (95% CIs) were reported. Multicollinearity assumption of the model was checked using the variance inflation factor (VIF) [16]. All analyses were conducted using IBM SPSS Statistics 28.0.

## Results

There were 6,665 respondents between 10 and 16 years old in the survey who reported their change in mental health status since the COVID-19 pandemic. Nearly half (n = 3,298, 49.5%) expressed that their mental health status was unchanged. More participants (30.8%) reported that they had poorer mental health than those who reported having better mental health (19.7%).

Descriptive statistics of the characteristics of respondents in different mental health status categories are summarized in Table 1. Statistically significant differences in all variables of interest were observed between the three categories. Students reporting better mental health since COVID-19 were more likely to be male, report satisfaction with academic performance, school life, relationship with classmates and family life, and have longer sleeping and exercise time (*p* < 0.001).

**Table 1:** Characteristics of respondents by self-reported mental health status since COVID-19

	Mental Health		
	Poorer	Unchanged	Better
	N = 2055	N = 3298	N = 1312
	Mean ± SD / N (%)	Mean ± SD / N (%)	Mean ± SD / N (%)
Age	14.42 ± 1.28	14.18 ± 1.28	14.30 ± 1.26
Sex (Male)	984 (47.9)	1816 (55.1)	814 (62.0)
Satisfaction with academic performance			
Dissatisfied	772 (37.6)	722 (21.9)	270 (20.6)
Neutral	980 (47.8)	1934 (58.7)	729 (55.6)
Satisfied	299 (14.6)	637 (19.3)	312 (23.8)
Satisfaction with school life			
Dissatisfied	147 (7.2)	111 (3.4)	61 (4.7)
Neutral	869 (42.3)	1263 (38.3)	416 (31.7)
Satisfied	1039 (50.6)	1922 (58.3)	834 (63.6)
Relationship with classmates			
Dissatisfied	56 (2.7)	47 (1.4)	29 (2.2)
Neutral	667 (32.5)	963 (29.3)	335 (25.6)
Satisfied	1331 (64.8)	2281 (69.3)	946 (72.2)
Satisfaction with family life			
Dissatisfied	221 (10.8)	123 (3.8)	50 (3.8)
Neutral	753 (36.8)	978 (29.9)	313 (24.1)
Satisfied	1074 (52.4)	2165 (66.3)	938 (72.1)
Average sleeping time in the past month			
Weekdays			
7–9 h	1719 (55.4)	812 (41.5)	672 (54.1)
< 7 h	1214 (39.1)	1063 (54.3)	481 (38.7)
> 9 h	169 (5.4)	82 (4.2)	90 (7.2)
Weekends			
7–9 h	1678 (54.4)	984 (50.5)	628 (50.6)
< 7 h	370 (12.0)	301 (15.5)	116 (9.4)
> 9 h	1038 (33.6)	663 (34.0)	496 (40.0)
Average exercise time in the past month			
Weekdays			
≥ 30 min	1382 (68.7)	2247 (70.2)	970 (75.5)
Weekends			
≥ 30 min	1320 (65.6)	2153 (67.1)	936 (72.6)

Note: All tests are statistically significant with a *p*-value less than 0.001

Table 2 reports differences in sample characteristics over categories of mental health status. The multicollinearity assumption was not violated in any model, with VIF less than 5 for all variables. Females reported overall poorer mental health post-pandemic compared to males. The odds of females having poorer mental health compared with unchanged was 36% (95% CI = 16-59%) higher than males, whilst the odds of having better mental health compared with poorer mental health was 28% (95% CI = 11-41%) lower for females than males.

**Table 2:** Results of multinomial logistic regression on the association between variables of interest and self-reported mental health status of respondents since COVID-19

	Mental Health *		
	Better vs. Unchanged (reference)	Poorer vs. Unchanged (reference)	Better vs. Poorer (reference)
	Odds Ratio (95% CI)	Odds Ratio (95% CI)	Odds Ratio (95% CI)
Age	1.048 (0.973–1.129)	1.048 (0.988–1.112)	1.021 (0.942–1.105)
Sex (Reference: male)	0.933 (0.768–1.133)	***1.355 (1.159–1.585)*** ^***a***^	***0.724 (0.587–0.893)*** ^***b***^
Satisfaction with academic performance (Reference: Neutral)			
Dissatisfied	0.963 (0.756–1.226)	***1.468 (1.233–1.748)*** ^***a***^	***0.637 (0.498–0.813)*** ^***a***^
Satisfied	1.020 (0.804–1.295)	1.096 (0.888–1.352)	0.935 (0.714–1.224)
Satisfaction with school life (Reference: Neutral)			
Dissatisfied	1.112 (0.655–1.889)	0.998 (0.679–1.468)	1.519 (0.918–2.513)
Satisfied	1.196 (0.944–1.514)	1.024 (0.850–1.233)	1.132 (0.882–1.453)
Relationship with classmates (Reference: Neutral)			
Dissatisfied	1.352 (0.640–2.852)	0.989 (0.559–1.748)	1.275 (0.630–2.582)
Satisfied	1.034 (0.807–1.326)	1.209 (0.997–1.466)	0.925 (0.712–1.201)
Satisfaction with family life (Reference: Neutral)			
Dissatisfied	1.564 (0.955–2.561)	***1.431 (1.040–1.970)*** ^***c***^	0.748 (0.473–1.185)
Satisfied	***1.261 (1.006–1.579)*** ^***c***^	0.850 (0.717–1.008)	***1.369 (1.085–1.728)*** ^***b***^
Average sleeping time in the past month			
Weekdays (Reference: 7–9 h)			
< 7 h	1.125 (0.909–1.391)	***1.475 (1.256–1.732)*** ^***a***^	0.827 (0.665–1.029)
> 9 h	1.101 (0.733–1.654)	1.057 (0.721–1.550)	1.028 (0.641–1.648)
Weekends (Reference: 7–9 h)			
< 7 h	0.794 (0.569–1.108)	1.078 (0.855–1.359)	***0.707 (0.504–0.992)*** ^***c***^
> 9 h	***1.316 (1.073–1.614)*** ^***b***^	1.098 (0.926–1.302)	1.232 (0.991–1.533)
Average exercise time in the past month			
Weekdays			
≥ 30 min (Reference: < 30 min)	1.008 (0.780–1.303)	0.967 (0.798–1.187)	1.094 (0.834–1.434)
Weekends			
≥ 30 min (Reference: < 30 min)	0.860 (0.667–1.108)	1.090 (0.892–1.333)	0.877 (0.665–1.029)
Number of observations	4,610	5,353	3,367
Overall significance	< 0.001	< 0.001	< 0.001
Nagelkerke R^2^	0.574	0.523	0.591

Notes: *: All the models controlled for depressive/anxiety symptoms and change in physical health status since COVID-19

^a^: *p* < 0.001, ^b^: 0.001 < *p* < 0.01, ^c^: 0.01 < *p* < 0.05

For school-related experiences, being dissatisfied with academic performance (with reference to neutral) increased the odds of having poorer mental health by 47% (95% CI = 23-75%) compared to having unchanged status. It also decreased the odds of having better mental health by 36% (95% CI = 19-50%) compared to having poorer status. However, being satisfied with academic performance was not related to mental health. No associations were observed between satisfaction with school life and relationship with classmates with mental health.

For sleeping time, those who slept less than seven hours per day during weekdays had higher odds of having poorer mental health compared with unchanged mental health status, compared to those who slept 7–9 h during weekdays. However, the difference was not statistically significant when comparing respondents with poorer and better mental health status. On the other hand, sleeping more than nine hours per day during weekends increased the odds of having better mental health with reference to unchanged status compared to sleeping 7–9 h during weekends, although the association was not statistically significant between respondents with poorer and better mental health status. No association was observed between exercise time and mental health status.

Being satisfied with family life was the only variable beneficial to mental health, increasing the odds of having better mental well-being post-pandemic by 26% (95% CI = 1-58%) and 37% (95% CI = 9-73%) compared to having unchanged or poorer status, respectively.

The experience of depressive/anxiety symptoms was used as the control variables in the multinomial regression analyses. Although not shown in table, the results indicated that respondents who felt nervous, anxious or on edge were less likely to report better mental health status with reference to poorer status compared to people with no such feelings.

## Discussion

The COVID-19 pandemic changed the daily routine of most young people in Hong Kong. This is one of the first studies to explore the perceived change in mental health status among young people pre-peri COVID-19, and to consider its association with school, family, and life-related factors. The findings suggested that approximately half the respondents reported unchanged mental health status pre-peri pandemic, and one-fifth of respondents indicated improved mental health. The proportion of adolescents who reported improved mental health was similar to that in Canada (19-31%) and higher than that in the US (5%) [8, 9]. This discrepancy may arise from the different surveying method and time. The study in the US was conducted three months after the beginning of the pandemic and the survey was completed by parents.

Regarding sex difference, poorer post-pandemic mental health was associated with being female. The result was consistent with the pre-pandemic findings in Hong Kong, where more depressive symptoms were reported among female adolescents [17, 18]. Researchers proposed that the sex difference in mental health status may be explained from the affective, biological and cognitive aspects [19]. International evidence also noted a considerable increase in the prevalence of major depressive and anxiety disorder among children and adolescents aged 10–19, and the increase was more prominent among females [20]. Future studies should be conducted to identify specific factors which resulted in the mental health disparities between sexes so as to provide evidence for the development of practical interventions.

This study also identified an association between dissatisfaction with academic performance and COVID-related mental health deterioration. However, the mental health status among respondents who reported being satisfied with academic performance did not differ pre-peri pandemic. This suggests that students struggling with academic performance may be more severely affected by COVID-19 than others. During the pandemic, conventional ways of schooling were no longer available due to school closures and lockdown measures, and most if not all academic activities became virtual. A recent review documented mixed results in the efficiency of online learning on academic performance [21]. The new learning mode may disproportionately influence those students who self-reported dissatisfying with their academic performance. However, school life satisfaction and classmates’ relationships were not associated with mental health status. This might also be explained by the virtual mode of learning during the pandemic, in which most students studied at home and had limited communication with their classmates.

The results provided evidence that some secondary students may have encountered difficulties with distance learning [22], especially those already struggling with pre-pandemic academic performance. Therefore, it is crucial for teachers, schools, and the government to collaborate to ensure students learn efficiently in the best way for them. Thus, special attention could be paid to students who encounter difficulties when new learning modes are introduced. In Hong Kong, although some of the pandemic-related policies have lessened and face-to-face teaching has been resumed in most schools, there is still occasional closure of schools for a few days to a week due to COVID-19 cases/clusters in school settings. On the findings of this research, teachers and parents should pay attention to students’ mental health status, particularly when they have additional needs. At the same time, the government should provide adequate support to teachers, schools, families, and students who experience difficulties in online study modes, such as those with limited Internet access and electronic devices. Apart from the students, other members in the family may face difficulties and mental distress because of the pandemic. Despite understanding learning difficulties, teachers and schools should also be aware of the needs of the extended family and refer them to support services, such as social workers and non-governmental organizations if necessary. Government should also provide support and allocate resources to help schools to connect with different services in order to assist the families in need.

Another important finding was that living through the pandemic provided an opportunity for improved mental health for some young people, within their family unit, as satisfaction with family relationships was strongly associated with improved mental health status post-pandemic. Family dynamics may have changed due to school closures, quarantine and lockdown, “work from home” policies, and the like during the pandemic [23]. Whilst the hybrid learning and working-from-home modes may have challenged some families, they may have increased opportunities for other families to spend more time together. It appears that quality family time has a positive influence on young people’s mental well-being. This finding supports the importance of promoting family relationships and increasing quality family time when improving mental health among young people, even after the pandemic has passed. Particularly, Hong Kong is among one of the most densely populated cities in the world with the highest living density [24]. Hong Kong is also one of the regions with the longest weekly working hours [25]. Efforts from corporations and the government will be required to provide family-friendly working environment. Increased time spent with family members, where there were harmonious relationships in constrained living spaces, may be beneficial to the mental well-being of young people.

### Limitations

This study has several limitations. First, it utilized cross-sectional self-reported survey data. Thus, the data are susceptible to recall bias and preclude establishing causal relationships between predictive factors and mental health status. Second, the survey may also not represent all Hong Kong secondary students, since non-Chinese speaking and evening schools were excluded. That said, only 6% of the day school students enrolled in international schools in 2020, and the number was much lower for evening schools [26].

## Conclusion

The COVID-19 pandemic changed the lives of most of the world’s citizens. The study showed that about 30% of Hong Kong young people perceived that they had poorer mental health status since the pandemic, although another 20% stated that their status improved. Females and those with dissatisfied academic performance were more likely to have poorer mental health status, while having satisfied family life was associated with improved mental health. Despite the gradual recovery, the pandemic situation remains volatile. While it is important to rigorously control the pandemic to protect the public from infection, it is essential to also protect the physical, mental and social well-being of young people in Hong Kong, as they are the generation who will carry the learnings and experiences of living through the pandemic into their adulthood. It is crucial to understand the factors associated with the pre-peri pandemic changes in mental well-being, so as to provide support to those in need.

## Electronic supplementary material

Below is the link to the electronic supplementary material.

Supplementary Material 1

## Data Availability

The data that support the findings of this study are available from the Family Planning Association of Hong Kong but restrictions apply to the availability of these data, which were used under license for the current study, and so are not publicly available. Data are however available from the authors upon reasonable request and with permission of the Family Planning Association of Hong Kong.
