# Correction: Risk and protective factors related to changes in mental health among adolescents since COVID-19 in Hong Kong: a cross-sectional study

**DOI:** 10.1186/s13034-023-00634-7

**Published:** 2023-06-30

**Authors:** Cheuk Yui Yeung, Vera Yu Men, Wendy W. Y. So, Daniel Yee Tak Fong, Mona Wai Cheung Lam, Derek Yee Tak Cheung, Paul Siu Fai Yip

**Affiliations:** 1 Department of Social Work and Social Administration, The University of Hong Kong, Pokfulam, Hong Kong, SAR China; 2 Hong Kong Jockey Club Centre for Suicide Research and Prevention, The University of Hong Kong, Hong Kong, SAR China; 3 School of Nursing, Li Ka Shing Faculty of Medicine, The University of Hong Kong, Hong Kong, SAR China; 4 The Family Planning, Association of Hong Kong, Hong Kong, SAR China

**Correction: Child and Adolescent Psychiatry and Mental Health (2023) 17:68** 10.1186/s13034-023-00622-x

Following publication of the original article [1], the authors identified errors in Table 1. The numbers and percentages for the variable “Average sleeping time in the past month” in the column Poorer and Unchanged should be swapped. The correct Table 1 has been presented with this correction.

**Table 1:** Characteristics of respondents by self-reported mental health status since COVID-19

	Mental Health		
	Poorer	Unchanged	Better
	N = 2055	N = 3298	N = 1312
	Mean ± SD/N (%)	Mean ± SD/N (%)	Mean ± SD/N (%)
Age	14.42 ± 1.28	14.18 ± 1.28	14.30 ± 1.26
Gender (Male)	984 (47.9)	1816 (55.1)	814 (62.0)
Satisfaction with academic performance			
Dissatisfied	772 (37.6)	722 (21.9)	270 (20.6)
Neutral	980 (47.8)	1934 (58.7)	729 (55.6)
Satisfied	299 (14.6)	637 (19.3)	312 (23.8)
Satisfaction with school life			
Dissatisfied	147 (7.2)	111 (3.4)	61 (4.7)
Neutral	869 (42.3)	1263 (38.3)	416 (31.7)
Satisfied	1039 (50.6)	1922 (58.3)	834 (63.6)
Relationship with classmates			
Dissatisfied	56 (2.7)	47 (1.4)	29 (2.2)
Neutral	667 (32.5)	963 (29.3)	335 (25.6)
Satisfied	1331 (64.8)	2281 (69.3)	946 (72.2)
Satisfaction with family life			
Dissatisfied	221 (10.8)	123 (3.8)	50 (3.8)
Neutral	753 (36.8)	978 (29.9)	313 (24.1)
Satisfied	1074 (52.4)	2165 (66.3)	938 (72.1)
Average sleeping time in the past month			
Weekdays			
7–9 h	812 (41.5)	1719 (55.4)	672 (54.1)
< 7 h	1063 (54.3)	1214 (39.1)	481 (38.7)
> 9 h	82 (4.2)	169 (5.4)	90 (7.2)
Weekends			
7–9 h	984 (50.5)	1678 (54.4)	628 (50.6)
< 7 h	301 (15.5)	370 (12.0)	116 (9.4)
> 9 h	663 (34.0)	1038 (33.6)	496 (40.0)
Average exercise time in the past month			
Weekdays			
≥ 30 min	1382 (68.7)	2247 (70.2)	970 (75.5)
Weekends			
≥ 30 min	1320 (65.6)	2153 (67.1)	936 (72.6)

All tests are statistically significant with a *p*-value less than 0.001
