# Supplementary material for: Risk and protective factors related to changes in mental health among adolescents since COVID-19 in Hong Kong: a cross-sectional study
Source: Child Adolesc Psychiatry Ment Health. 2023 Jun 12;17:68. doi: 10.1186/s13034-023-00622-x (PMC10258769; doi:10.1186/s13034-023-00622-x)
Supplement: Supplementary file 1 — Supplementary Material 1 [file 13034_2023_622_MOESM1_ESM.docx]

Appendix I. Daily number of confirmed COVID-19 cases in Hong Kong, 1/23/2020-12/31/2022.

Notes: Since 3/9/2022, the daily confirmed case was the sum of “number of cases tested positive for SARS-CoV-2 virus by nucleic acid” and “number of cases tested positive for SARS-CoV-2 virus by rapid antigen tests” [3].

Appendix II. Daily number of confirmed COVID-19 cases in Hong Kong, 2021.

**Data collection period: May to July 2021**
